# Developing Future Public Health Leaders Trained in Long-term Care Administration

**DOI:** 10.1097/PHH.0000000000001188

**Published:** 2020-04-20

**Authors:** N. Ruth Gaskins Little, Polly Welsh, Adam Sholar

**Affiliations:** Department of Public Health, Brody School of Medicine, East Carolina University, Greenville, North Carolina (Dr Little); and North Carolina Health Care Facilities Association, Raleigh, North Carolina (Ms Welsh and Dr Sholar).

**Keywords:** education, leadership, long-term care, public health workforce

## Abstract

America's population is rapidly aging. Long-term care leaders have not been considered part of the public health workforce. The enumeration of long-term care into the public health workforce taxonomy has not occurred. This article examines the extent of graduate public health education oriented to long-term care and provides a case study of a successful curriculum at East Carolina University (ECU).

**Method:**

Web sites displaying the curriculum of 135 graduate programs/schools accredited by the Council on Education for Public Health (CEPH) were analyzed for graduate long-term care orientation. A case-study approach was used to describe the integration of long-term care into the Master of Public Health (MPH) Health Policy Administration & Leadership concentration at ECU.

**Results:**

A review of 135 CEPH graduate MPH programs from January to July 2019 found that only 8 institutions offered graduate courses in long-term care administration. Of the 8, ECU Brody School of Medicine Department of Public Health was the only program directly linking coursework to licensure as a long-term care administrator. Program graduates total 30, which include 5 MPH students currently completing their Administrator in Training. At time of graduation, 17 students had obtained North Carolina licensure.

**Conclusions:**

Because of increases in population aging, this requires a public health workforce with skills and training in the care of older adults. Formal recognition of long-term care workers as an integral part of the public health workforce is needed. The Institute of Medicine called for this action more than a decade ago.

The public health system is defined as “all public, private and voluntary entities that contribute to the delivery of essential public health services within any jurisdiction.”^1^ Improving the health of communities by reducing health disparities prevalent in underserved lower socioeconomic groups is an important focus of public health. Public health recognizes how social determinants of health affect the distribution of health for populations and advocates for policy changes to improve access to health care.^2^ One of the greatest public health policy achievements in the 20th century in the United States was the passage of Title 19 to the Social Security Act enacting the Medicaid program in 1965.^3^ A Kaiser (2017) report finds that 1 in 3 Americans will need nursing home care at some point in his or her lives, with Medicaid paying for the care of 6 of every 10 patients.^4^ Nursing homes are considered an integral part of the public health system.^5^,^6^ Although two-thirds of nursing home facilities in the United States are for-profit, these private sector providers are also considered a component of the public health system.^1^,^7^

Ensuring the health of communities is a vital role of the public health workforce.^8^ Yet, more than two-thirds of this workforce in the United States lacks formal education/training in public health. Of the one-third with formal public health training, many report the need for management training.^9^

The current COVID-19 pandemic and its effect on elderly persons, particularly those residing in skilled nursing facilities, imperil a subset of one of our most fragile populations.^10^ Consequently, the Centers for Disease Control and Prevention (CDC) has provided explicit policy guidance to nursing homes that requires the immediate classification and management of sick patients while also stressing the imperative for prevention education.^11^ A strong leadership response from nursing home administrators is critical for managing situations of this global magnitude. Inclusion of long-term care as a vital element of the US public health workforce is the basis for the program described in this article. The nationwide deficit in public health training for long-term care workers parallels or exceeds those in other areas of public health. This article presents a successful model for training licensed long-term care administrators who are also Master of Public Health (MPH) graduates trained in prevention, data management, health policy, administration, and leadership.

## Background

Individuals 65 years and older will constitute 20% of the total US population by 2030. By 2035, it is projected that there will be 78 million people 65 years and older compared with 76.7 million of those who are younger than 18 years.^12^ This means that for the first time in the history of the United States, people 65 years and older will constitute a greater percentage of the total US population than those 18 years and younger. The unprecedented growth in our senior population will increase demand for services not only from hospitals but also from our skilled nursing homes and residential long-term care facilities.^13^ In 2016, hospital, nursing home facilities, and home health care comprised 47.2% of total health care expenditures. Skilled long-term care facilities, also known as “nursing homes,” are primarily for-profit (69.3%) providing care for more than 1.3 million residents in 2015.^7^,^14^

Nursing home administrators are the designated leaders of their facilities and are required to hold a federal license.^15^ Many states also require a state license, but not all. Variation exists in the minimum eligibility age for licensure as well as educational degree, length, and duration of Administrator in Training (AIT), as well as the number of hours required for licensure and continuing education requirements. Of note is the fact that 19 states require either no education beyond high school or GED in order to be eligible to apply to become a nursing home administrator.^16^ See Table 1.

**TABLE 1:** Nursing Home Administrators Licensure Requirements Summary^a^

Licensure Requirements				AIT Training		Examination		CE Hours Required
State	Minimum Age	Education						
		Degree	Min/Max Hours	Period	Board-Approved Preceptor	National	State	
Alabama	19	AA		200/2000	Yes	Yes	Yes	24/Annually
Alaska	19	BA				Yes	No	0/0
Arizona		BA		1000	No	Yes	Yes	50/Biennially
Arkansas	21	AA				Yes	Yes	20/Annually
California NHA	18	BA		1000	Yes	Yes	Yes	40/Biennially
Colorado	21	AA		500/1000	Yes	Yes	Yes	0
Connecticut		0				Yes		0
Delaware	18	Other	100		Yes	Yes	No	48/Biennially
District of Columbia	18	BA		1000	Yes	Yes	Yes	40/Biennially
Florida	18	BA		1000/2000	Yes	Yes	Yes	40/Biennially
Georgia								
Hawaii	21	BA	30	2080/0	Yes	Yes	No	0/Biennially
Idaho NHA	21	BA		384/384	Yes	Yes	Yes	20/Annually
Illinois		0	1/1	/1	Yes	Yes	Yes	/0
Indiana		Other	280	728/1040	Yes	Yes	Yes	40/Biennially
Iowa		BA	12	720	No	Yes	No	40/Biennially
Kansas	18	BA		480	Yes	Yes	Yes	50/Biennially
Kentucky	21	BA		1080	No	Yes	No	30/Biennially
Louisiana	21	BA		1040	Yes	Yes	Yes	15/Annually
Maine		0						0
Maryland	21	BA	100/100	2080/2080	Yes	Yes	Yes	40/Biennially
Massachusetts	18	BA		520/1040	Yes	Yes	No	40/Biennially
Michigan	18	Other	0/0	0/0		Yes	Yes	36/Biennially
Minnesota	21	BA	0/36	0/400	No	Yes	No	20/Annually
Mississippi	21	Other	0/0	1040	Yes	Yes	Yes	40/Biennially
Missouri	21	Other		500/2000	Yes	Yes	Yes	40/Biennially
Montana	18	Other				Yes	Yes	25/Annually
Nebraska	19	AA		640	Yes	Yes	No	50/Biennially
Nevada	21	BA		1000	Yes	Yes	No	30/Biennially
New Hampshire	21	BA	15		No	Yes	Yes	40/Biennially
New Jersey	18	BA	100/100	1750/1750	Yes	Yes	No	60/0
New Mexico		BA			No	Yes	Yes	24/Annually
New York	21	BA	15	445/2080	Yes	Yes	No	48/Biennially
North Carolina	18	Other	0/0	480/2000	Yes	Yes	Yes	30/Biennially
North Dakota	18	BA	0	480	Yes	Yes	Yes	20/Annually
Ohio	18	BA		1500/1500	Yes	Yes	Yes	20/Annually
Oklahoma	21	BA		560/560	Yes	Yes	Yes	24/Annually
Oregon		BA		960	No	Yes	Yes	20/Annually
Pennsylvania	21	Other		800/1000	No	Yes	Yes	48/Biennially
Rhode Island	18	BA	15	350		Yes	No	40/Biennially
South Carolina	21	BA	0/0	0/0	Yes	Yes	Yes	20/Annually
South Dakota	18	BA		240	Yes	Yes		40/Biennially
Tennessee	18	AA		400	Yes	Yes	Yes	18/Annually
Texas		BA	15	1000	Yes	Yes	Yes	40/Biennially
Utah		0		1000	No	Yes	No	40/Biennially
Vermont	18	BA	0/0	1000	Yes	Yes	Yes	40/Biennially
Virginia		BA		320/2000	Yes	Yes	No	20/Annually
Washington		BA		1500	Yes	Yes	No	36/Biennially
West Virginia	21	BA	12	2040	Yes	Yes	Yes	20/Annually
Wisconsin		0						0
Wyoming		BA			No	Yes	No	25/Annually

Abbreviations: AA, Associate of Arts; BA, Bachelor of Arts; NHA, nursing home administrator.

^a^From National Association of Long Term Care Administrator Boards (https://www.nabweb.org/state-licensure-requirements).

The classification and enumeration of the public health workforce have been difficult to quantify.^17^ The University of Michigan Center of Excellence in Public Health Workforce Studies convened national experts in 2013 to define the public health workforce by 287 classifications across 12 domains (axes) providing definitive job classifications including occupational setting and employment. Under Axis 2: Local Setting, hospitals are listed as a local public health setting but long-term care facilities were not included.^17^ Yet, nursing homes in 2019 constituted twice the number of health care facilities as hospitals. There are more than 15 600 nursing homes in the United States comprising 1.7 million licensed beds caring for more than 1.3 million residents.^7^ This compared with 6 210 hospitals totaling 931 203 staffed beds with 36 510 207 admissions.^18^

The Public Health and Welfare United States Code, Title 42, 42 USC §1396g, requires federal licensure for nursing home administrators.^15^ Axis 1: Management and Leadership does list public health manager as an occupation, but long-term care or nursing home administration is not listed in Axis 9 as an area of expertise or under 1.1.12 Licensure/Regulation/Enforcement worker. Axis 1: Management and Leadership (1.11-1.1.6) is the only occupational reference that aligns with traditional federal/state public health positions.^17^ The Institute of Medicine (IOM) in 2008 pointed out an urgent need to improve training and competency of the eldercare workforce, with a specific challenge to public health to enhance the skills of any health care worker/practitioner involved in providing care to elderly persons.^19^ Despite 42 USC §1396g^15^ being contained under the US Public Health code, leaders of long-term care facilities are disadvantaged by the lack of enumeration of their employees in the public health workforce. The purpose of this article is to describe a successful collaboration between academic practice and long-term care industry leaders to formally integrate graduate public health education with long-term care administration, leading to graduates attaining licensure as long-term care administrators. The East Carolina University (ECU) program serves as a model for other institutions.

## Methods

A list of accredited graduate programs in public health was obtained from the Council on Education for Public Health (CEPH).^20^ Each MPH program's curriculum was reviewed for any graduate courses oriented to long-term care by reviewing institutional Web sites (and graduate catalogues if available) for degree and course information. A database was created in Excel by the authors containing the name of the institution, degree, course number, and title of courses oriented to long-term care. The purpose of creating the database was to determine the prevalence of graduate-level coursework in long-term care administration in CEPH-accredited MPH programs. Institutional Web sites and content were reviewed to determine whether any narrative language existed at the program or course level that described the integration of graduate MPH coursework as leading to federal/state licensure as a nursing home administrator.

A case-study approach was used to describe the program concentration at the ECU MPH program. The faculty and advisors to the program were interviewed including input from long-term care industry executives, members of the North Carolina state licensing board, licensed administrators, and the director of the Eastern Area Education Center. Meeting notes, e-mails, AIT information, as well as follow-up interviews were conducted of students participating in this concentration.

## Results

A review of CEPH MPH graduate programs and schools of public health from January to July 2019 found that only 8 institutions had graduate courses in long-term care administration. Two of the 8 institutions, the University at Albany-SUNY and New York Medical College, each offered 2 courses in long-term care. Oregon State University School of Public Health offered 1 graduate MPH course in long-term care. ECU Department of Public Health at Brody School of Medicine offers 3 courses oriented as part of the focus on long-term care in the MPH Health Policy Administration & Leadership (HPAL) concentration. The University of Nevada, Las Vegas, offers 1 long-term care course in its School of Public Health Master of Health Administration (MHA) program. The University of North Florida offers 2 graduate courses in the Brooks College of Health as part of the MHA degree. The University of Alabama at Birmingham has a graduate course in long-term care as part of its Master of Science in Health Administration (MSHA) degree; the University of Minnesota has a long-term care course as part of a postbaccalaureate certificate in its school of public health. Of the 8 institutions, ECU Brody School of Medicine Department of Public Health was the only program that had coursework directly linked to licensure. See Table 2.

**TABLE 2:** Graduate Courses by CEPH-Accredited Institution

Institution	Course Title	Course Number	Location	Part of MPH	Lead to Licensure?
UAB	Long Term Care Administration	HA643	School of Health Professions	No	No
University of North Florida	Long Term Care Administration	HSA 6225	Brooks College of Health	No, MHA	No
University of North Florida	Long Term Care Internship	HAS 6945	Brooks College of Health	No, MHA	No
University of Minnesota^a^	Long Term Care Principles, Programs & Policies	PUBH 8803	School of Public Health	No, postbaccalaureate certificate	No
University of Nevada, Las Vegas	Organization & Management of Long-Term Care Services	HCA 680	School of Public Health	MHA	No language stating leading to licensure
University at Albany-SUNY	Managing Long Term Care Services	HPM 528	School of Public Health	Yes, MPH in Health Policy, Management & Behavior	No language regarding administrator licensure
University of Albany-SUNY	Long Term Care Administration	HPM 643	School of Public Health	Yes, MPH in Health Policy & Management	No language stating leading to licensure
New York Medical College	Managing Long Term Care Facilities	HPMM 6061	School of Health Sciences & Practice & Institute of Public Health	Yes, MPH in Health Policy & Management	No language stating leading to licensure
New York Medical College	Long-Term Care Delivery Systems	HPMM 6029	School of Health Sciences & Practice & Institute of Public Health	Yes, MPH in Health Policy & Management	No language stating leading to licensure
East Carolina University	Long Term Care Administration	MPH 6040	Brody School of Medicine	Yes, MPH HPAL concentration	Yes
East Carolina University	Field Practicum	MPH 6903, 6904/6905	Brody School of Medicine	Yes, MPH HPAL concentration	Yes, students focusing in long-term care administration take an additional field practicum course due to length of AIT counting as an elective
Oregon	Financing & Administration of Long-Term Care Facilities	H568	College of Public Health & Human Sciences	Yes, MPH Health Management & Policy	No language regarding licensure, course offered as elective

Abbreviations: AIT, Administrator in Training; CEPH, Council on Education for Public Health; HPAL, Health Policy Administration & Leadership; MHA, Master of Health Administration; MPH, Master of Public Health.

^a^Postbaccalaureate certificate only.

## Case Study: Development of a Focus on Long-term Care Administration at ECU MPH Program

The integration of long-term care administration into the MPH degree was accomplished by the successful collaboration between research and practice. This initiative was led by the Director of Field Placement for the MPH program, who had previous experience both as a licensed long-term care administrator and a local public health director, who with department chair support convened health care leaders in practice and in academia including the president and vice president of the North Carolina Health Care Facilities Association, the president of the North Carolina Hospital Association, long-term care industry executives, the executive director of the North Carolina Board of Examiners for Nursing Home Administrators, a representative from the North Carolina Department of Regulatory Services Administration, licensed administrators, and the director of the Eastern Area Education Center. This work group, in partnership with ECU faculty, helped design the curriculum that was ultimately added as a focus within the HPAL concentration in the MPH program at ECU's Brody School of Medicine. A new course in Long Term Care Administration was developed with input from the work group for developing the course objectives. Work group members frequently provide guest lectures on their areas of expertise.

## Program Requirements

The MPH HPAL concentration consists of 45 hours of coursework including 24 credit hours of core classes encompassing public health foundational coursework. The long-term care focus requires 9 credit hours of focus courses, 6 credit hours of electives, a 3-credit internship, and a 3-credit professional paper.^21^ An individual seeking to become a licensed nursing home administrator in North Carolina must complete an AIT requirement prescribed by the North Carolina Board of Examiners for Nursing Home Administrators based on education and experience.^22^ Because of the length of the AIT, MPH students seeking licensure fulfill a rigorous field placement to prepare them for state and federal licensure and may take a second internship course as an elective. Students typically sit for their state and national examinations near their graduation.

## Program Funding for Sustainability

In 2018, the successful partnership to integrate long-term care administration into the MPH program resulted in the establishment of the J. Craig Souza Endowed Public Health Scholarship at Brody School of Medicine. The scholarship supports MPH students who choose to train to become licensed administrators by providing financial support during their AIT licensure requirement and field placement courses. Scholarship funding was derived from donations by long-term care industry leaders including Mr Souza.

## Program Achievements

There are now 25 alumni of the long-term care focus within the MPH program. An additional 5 students are completing their AIT, with expected graduation May 2020. Twenty-one are female and 9 are male students. Three alumni were long-term care professionals, 2 licensed administrators, and 1 an industry executive before completing their MPH degrees. Seventeen were licensed in North Carolina while completing their MPH degree (10 females and 7 males). Of those licensed administrators with an MPH degree, 6 are African American, 2 are Native American, 8 white, and 1 from India. Five students are currently completing their AIT in the second year of their MPH degree; all 5 are female, with 1 Hispanic, 1 African American, 1 from India, and 2 White. Four MPH licensed administrator alumni have completed additional training to become certified preceptors in the concentration and are precepting current MPH students. See Table 3.

**TABLE 3:** MPH HPAL Student Demographic and Licensure/Preceptor Data

Variables	Total, N^a^	Long-term Care Professional Completing MPH	Current MPH Students Seeking Licensure	Licensed While Enrolled in MPH	MPH But Not Licensed	MPH LNHA Also Preceptors
Female	21	2	5	10	4	1
Male	9	1	0	7	1	3
Black	9	0	1	6	1	1
Hispanic	1	0	1	0	0	0
White	15	3	2	8	3	3
Native American	2	0	0	2	0	0
Other	3	0	1	1	1	0
Total, N	30	3	5	17	5	4

Abbreviations: AIT, Administrator in Training; LNHA, Licensed Nursing Home Administrator; MPH, Master of Public Health.

^a^Includes 5 AITs currently completing their AIT.

## Continuing Education of the Long-term Care Workforce

The assembled work group identified another compelling need: to develop and implement a training continuum for the current long-term care workforce targeted to individuals with associate degrees in nursing so they could obtain their BSN degrees online through the ECU College of Nursing. They also recommended that individuals with non-nursing associate degrees employed in the long-term care workforce could first obtain their bachelor's degree in Health Services Information Management online at ECU and then proceed to enroll in the MPH program.

This work group also articulated the need for ensuring that the future long-term care workforce had training and expertise in public health. This initiative led to successful funding from the Duke Endowment, demonstrating the strength of partnerships and philanthropic commitment for preparing current and future long-term care administrators with graduate education in public health. The objective of the Duke Endowment Long-Term Care Training Continuum proposal was to develop an educational multistep program responsive to the needs and challenges of those seeking training in long-term care administration at ECU. Grant funding supported individuals with associate degrees working in long-term care to obtain their baccalaureate degree in either nursing or health services management and subsequently their MPH degree. Continuing education required for licensure maintenance is provided by Area Health Education Centers.

## Discussion

While some health care leaders hold an MHA degree,^23^ distinct differences exist between the MHA and MPH curricula. The MHA curricula is more financial/business management focused and often lacks curricula attention to public organizations inherent in public health coursework and practice.^24^,^25^ The MPH in HPAL includes foundational public health coursework content in biostatistics, epidemiology, environmental health, health policy administration and leadership, health behavior, research methods/data management, disaster preparedness in addition to concentration coursework in financial management, health administration/leadership, and health policy focusing on reducing health disparities by improving population health understanding of the importance of social determinants of health.^21^ In fact, the accrediting body for public health, the CEPH, has specific MPH competencies that must be obtained before degree conferral that includes planning, management, and leadership competencies.^26^

The Council on Linkages Between Academia and Public Health Practice has identified core competencies for public health professionals with MPH degrees by tier levels (1-3), with tier 3 competency attainment the expectation for leaders of health care organizations.^27^ Nursing home administrators are the designated leaders of their facilities as required by licensure.^15^ The CDC's COVID-19 directives for nursing homes require systems management and monitoring for early detection and management of diseased patients, surge capacity planning, human resources staffing, and fiscal evaluation and compliance.^11^ Superlative leadership skills are required of nursing home administrators who must coordinate with multiple local, state, and federal agencies, as well as the onslaught of media and family inquiries during this pandemic. Yet, current educational requirements for licensure of nursing home administrators do not require these skills.

The long-term care focus in the HPAL concentration is critically important for the ongoing vitality of the health care workforce and to ensure the health of the US population. The program has increased the number of long-term care administrators with a public health education. Administrators with an MPH degree can apply their leadership, policy, and management skills through a public health lens. They understand the importance of prevention and are equipped with skills to not only deter outbreaks but also reduce the spread of exposure by effective management and enactment of isolation and quarantine measures when needed. Nursing home administrators with an MPH degree are equipped to navigate the totality of the health care system. They understand the role and function of the many agencies under the public health umbrella and have been trained to be effective communicators and managers to respond appropriately and effectively to pandemics as we are now experiencing worldwide. In addition, the long-term care focus is fulfilling the IOM's 2008 mandate to train more of the eldercare workforce in public health.^19^ With this expansion has come the availability of more preceptors with MPH degrees to train future administrators grounded in public health principles, equipped and able to respond to future forms of public health disasters.

Women have lower odds of achieving executive-level leadership positions despite constituting a majority of the public health workforce.^28^ The long-term care focus within the HPAL program is increasing the number of women and minorities in public health leadership positions. Recognizing the concern arising from the lack of connectivity between educational degrees and workforce skills^29^ and also student loan debt, it is important to note that 16 of the 17 licensed administrators received and accepted offers for employment as long-term care administrators by the time of their MPH graduation.

**Implications for Policy & Practice**

- Study findings indicate a need to increase the health care workforce equipped with public health prevention skills to care for our elderly population that utilizes long-term care services.^30^
- Formal recognition of long-term care as an integral part of the public health workforce is critically needed to ensure that long-term care leaders are trained in public health with the skills and competencies to facilitate interprofessional health care delivery as demanded by our health care system design.

## Conclusion

The COVID-19 pandemic brings heightened awareness to the demands upon the health care workforce exacerbated by America's rapidly aging population. We have entered a new arena that requires public health workforce leaders to have the skills and abilities to respond to not only social and environmental problems but also microbial, biological, and physiological diseases. The partnership between academia and the leadership of the long-term care industry in North Carolina resulted in the integration of a focus on long-term care administration into the HPAL concentration for the MPH degree at ECU. Importantly, this degree leads to state and federal licensure as a nursing home administrator and serves as a model for adoption by other graduate public health programs.
